# 

**Published:** 1891-03

**Authors:** 


**The Mississippi Valley Medical Association** will hold its seventeenth annual session at St. Louis, Wednesday, Thursday and Friday, October 14, 15 and 16, 1891. A large attendance, a valuable program and a good time are expected. The members of the medical profession are respectfully invited to attend. C. H. Hughes, M. D. President, 500 N. Jefferson Avenue, St. Louis; E. S. McKee, M. D., Secretary, 57 West Seventh Street, Cincinnati; I. N. Love, M. D., Chairman Committee of Arrangements, 301 N. Grand Avenue, St. Louis.

**The Waco Meeting.**—Let it be borne in mind that the 28th annual session of the Texas State Medical Association will meet at Waco, beginning Tuesday, April 28th and holding four days. Applicants for membership *must present diploma* with application; and having previously registered and paid the Treasurer (who will be on the stand in advance of meeting) $10, pin his receipt to application also, and hand it to the Secretary; it will then go to the Judicial Council for action.

---

**Let it be remembered** by those who will attend the Waco meeting that payment of dues is absolutely imperative. The Treasurer will have charge of the badges, and will deliver them with his receipt on payment of dues.

---

**The Secretary** again calls on the hundred and odd members who have not settled with the Treasurer for last year, to commumunicate with him at once, and a card from him to the Secretary will secure a copy of the Transactions of '90. Thsse copies are wrapped and addressed to over a hundred members, and are lying in the Secretary's office, and he is prohibited from mailing them except on instructions from the Treasurer. Fourth notice. Don't blame the secretary for doing his duty.

DANIEL'S TEXAS MEDICAL JOURNAL is *sui generis;* it is a departure from the stereotyped form of journals which contain nothing but medicine, surgery, prescriptions, etc.; it is a *medical magazine*—a medical newspaper,—and contains more original matter than any journal published. It comments on medicine, medical politics and medical news, etc. Every Texas physician should take it. It is devoted to organizing the medical profession that we may obtain needed reforms, and advance both medical and sanitary science. Get your friends to subscribe. Clubs of three or more at 25 per cent. discount. Begin now; this is an excellent number.

